# Public engagement with research reproducibility

**DOI:** 10.1371/journal.pbio.3002953

**Published:** 2024-12-11

**Authors:** Constant Vinatier, Magdalena Kozula, Veerle Van den Eynden, Laura Caquelin, Hynek Roubik, Inge Stegeman, Florian Naudet

**Affiliations:** 1 Univ Rennes, CHU Rennes, Inserm, EHESP, Irset (Institut de recherche en santé, environnement et travail) - UMR_S 1085, Rennes, France; 2 Faculty of Psychology and Educational Sciences, Methodology of Educational Sciences Research Group, KU Leuven, Leuven, Belgium; 3 RDM Competence Centre, KU Leuven, Leuven, Belgium; 4 Department of Clinical Neuroscience, Karolinska Institutet, Stockholm, Sweden; 5 Department of Sustainable Technologies, Faculty of Tropical AgriSciences, Czech University of Life Sciences, Prague, Czech Republic; 6 Department of Otorhinolaryngology and Head & Neck Surgery University Medical Center Utrecht, Utrecht, the Netherlands; 7 Brain Center, University Medical Center Utrecht, Utrecht, the Netherlands; 8 Institut Universitaire de France (IUF), Paris, France

## Abstract

Public engagement with reproducibility is crucial for fostering trust in science. This Community Page describes how, by using relatable analogies and hands-on activities, participants explored the challenges of research replication, highlighting the importance of transparency and clear methods in the scientific process.

## Teaser

Public engagement with reproducibility is crucial for fostering trust in science. This Community Page outlines, through the example of baking Christmas tree meringues, how scientists can effectively engage and educate the public about the importance of reproducibility in research.

## Introduction

Trust in science is vital for society, as public understanding plays a crucial role in the acceptance and implementation of scientific knowledge. Research reproducibility is a fundamental component of maintaining this trust; it helps to identify mistakes and is crucial for transparency. However, there are growing concerns that numerous studies across many fields prove difficult or impossible to replicate [1]. Various factors, including reporting bias, small sample sizes, and questionable research practices like p-hacking—where researchers repeatedly test data in different ways to find significant results [2]—contribute to this troubling reality. Irreproducible research not only impedes academic progress, but it may also exacerbate misplaced skepticism when communicated to the public. This situation creates a paradox: increasing public literacy on the complexities of reproducibility could foster a more nuanced understanding of research results, but also risks further eroding confidence in science. It is therefore crucial that this topic can be communicated to the broader public in an accessible, positive, and constructive manner.

## Challenges in communicating (ir)reproducibility

Science communication is all too often polarized, with one side promoting scientific discoveries in an exaggerated manner without adequately addressing their limitations [3,4], while the other reports on a lack of reproducibility in science. Stories on the latter are frequently sensationalized, with narratives such as “science is broken” or news stories focusing heavily on instances of fraud and scientific misconduct [5,6]. Even within the scientific community, prestigious outlets sometimes push the narrative of a widespread “reproducibility crisis” [1], though researchers lack consensus on its existence [7]. These narratives may obscure the complexities and nuances of the scientific process. A more effective communication about research reproducibility should strike a balance by acknowledging both the challenges and controversies in science without eroding trust in its methods. Discussions should rather highlight the inherent uncertainty and variability of scientific experiments and the crucial role reproducibility plays in responsible research [8]. By providing context and explaining the significance of reproducibility and rigorous methodologies, one can demystify the research process and foster a deeper public understanding of how science works. These detailed descriptions, however, introduce a challenge in itself. It makes the way researchers communicate very technical. Research reproducibility is indeed a multifaceted concept that encompasses methods reproducibility (sufficient details are provided to reproduce the methods), results reproducibility (replication of results using the same methods as initial researchers did), and inferential reproducibility that brings interpretation of the findings into the mix [9]. However, understanding each of these facets requires a background in methods and statistics.

## Finding innovative ways to popularize reproducibility

Despite such challenges, addressing the topic of research reproducibility is far from destined to fail. Within the scientific community, “ReproducibiliTea” meetings have been highly successful among early-career researchers as a platform for discussing research reproducibility [10]. Their success is largely due to their focus on practical examples that resonate with researchers, who frequently encounter reproducibility issues in their daily work. These discussions are conducted in an educational manner, fostering a constructive environment without shaming. Another creative initiative for scientists is a LEGO-based game developed by the University of Glasgow to teach concepts of metadata and reproducibility, drawing parallels between describing the construction of a LEGO model and recording research methods in detail [11]. For the general public, the challenge lies in finding relatable examples, as most laypeople are not accustomed to replicating scientific experiments. Actually, many aspects of everyday life are subject to variability and replication challenges. Activities like drawing a picture, playing a musical piece, even cooking a recipe often produce inconsistent and occasionally disappointing results, making them powerful analogies to help the public grasp the challenges scientists face. Importantly, this approach aligns closely with well-established educational strategies using teaching-with-analogies models [12]. By using relatable examples, both public engagement and comprehension of scientific principles can be enhanced, bridging the gap between abstract concepts and everyday understanding.

**Fig 1:**
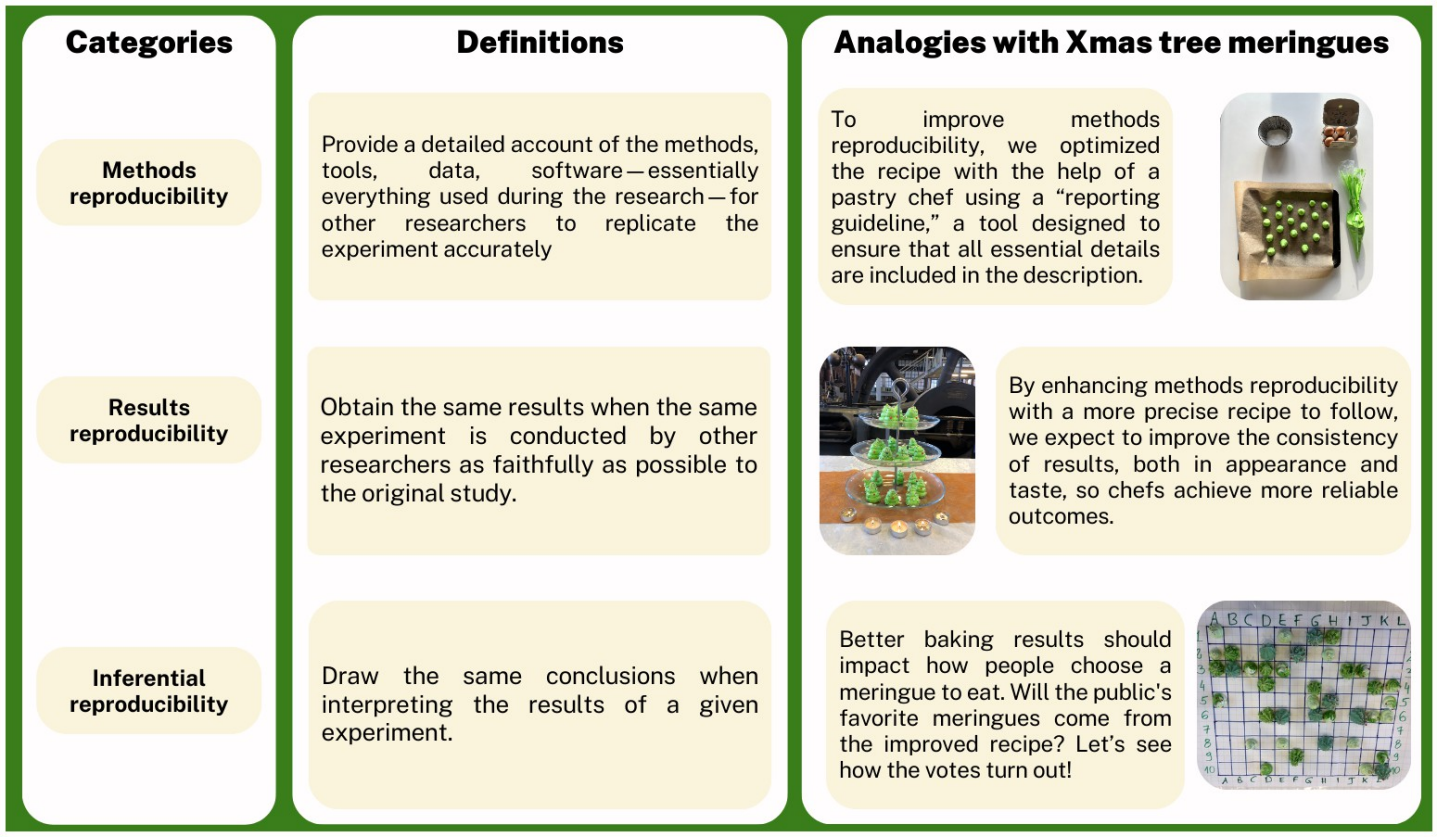
Definitions of types of reproducibility. Definitions for methods reproducibility, results reproducibility, and inferential reproducibility [9]. The analogy of Christmas tree meringues is given as an example to help to illustrate how reproducibility applies to research process outcomes and conclusions.

This is exemplified in the outreach by Open Science to Increase Reproducibility in Science (OSIRIS), a European-funded network focused on reproducible research practices, which organized 2 public events to promote these concepts using the analogy of baking Christmas tree meringues (Fig 1). In these events, volunteers were tasked with baking meringue Christmas trees, either following a standard recipe or a recipe enhanced by a baker with a reporting guideline—a checklist designed to improve reproducibility through added detail—and an instructional video. The first event took place during the 2023 Science Day (Dag van de Wetenschap) in Leuven, Belgium, where 8 teams of family and friends participated in “Bake off Science” [13]. The quality of their meringues was then evaluated blindly against a “gold standard” by judges posing as Michelin-starred chefs. A second event in Rennes, France, expanded the concept with 60 participants—both laypeople and medical students—who baked nearly 900 meringues [14] as part of a randomized controlled trial comparing the classic recipe with the improved recipe and instructional video. After a public evaluation of reproducibility by a blinded jury, all meringues were sold during a large public event. In addition to being the statistical units of the study, the meringues also served as a key attraction, drawing a diverse audience and increasing engagement.

**Fig 2:**
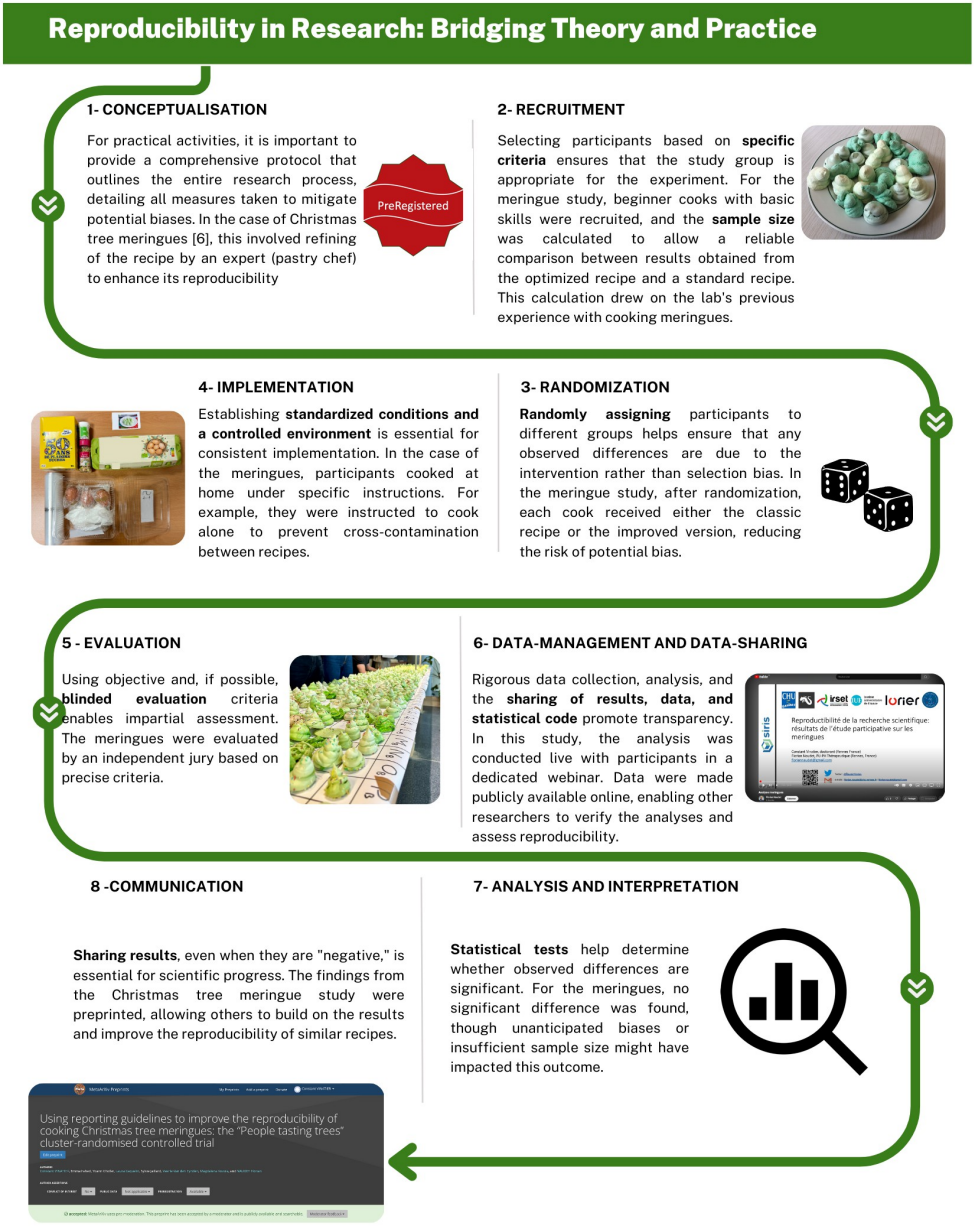
Communicating reproducibility in research with the public. A recipe for engaging and educating the public about reproducibility in science, including important considerations for planning, implementing, analyzing, and communicating effectively.

Despite the varied outcomes in visual appeal, color, size, taste, and sales, no significant improvements were found between the groups. Most meringues deviated from the ideal result. While the findings failed to demonstrate the superiority of the improved recipe, the experiment’s methods, including preregistration and data sharing, were made visible to participants, illustrating reproducible research practices (Fig 2). This approach effectively communicated the importance of research transparency. Moreover, negative results observed in the study sparked meaningful discussions about the importance of publishing such outcomes. This highlighted that reproducibility is not solely about producing identical results, but about ensuring that the research process is transparent, well-documented, and capable of being scrutinized or reproduced by others.

### How to effectively communicate the take-home messages to the public

These events illustrate that public engagement with science can be greatly enhanced through practical, relatable examples. By using hands-on activities, participants intuitively engaged with scientific concepts instead of relying on abstract explanations. At various levels, they grasped that the challenges of reproducibility in daily life mirror the difficulties of achieving consistent results in scientific experiments. They encountered firsthand the difficulties in replicating a result and reflected on the numerous variables that could lead to outcomes far from the ideal recipe, akin to the degrees of freedom researchers attempt to control through experimental methods.

Several lessons for future initiatives can be drawn from these 2 events. Tailoring the analogies with reproducibility of research to the audience and the context of the event is crucial. Balancing scientific rigor with engaging and accessible content requires careful curation of content. Some participants may not fully grasp the deeper scientific implications of the analogies. Hence, facilitators must adapt their explanations to a diverse audience, spanning different ages, educational levels, and scientific backgrounds, e.g., by using different means to facilitate communication such as accompanying posters addressing this potential diversity.

Since the 2 events attracted some media coverage, it seems important that facilitators receive appropriate media training beforehand. This training should equip researchers with essential skills to clearly communicate complex topics in an approachable and relatable way to a diverse audience and to discuss uncertainties openly. Additionally, it should help them in avoiding sensationalism by responding to misinterpretations and engaging effectively with the press and public by handling interviews and addressing public skepticism constructively. It indeed seems important to convey the idea that science is not broken, but rather that scientific research is a human activity with potential for biases and that the scientific approach aims to correct—as far as possible—these biases. And while some of the meringues may have resembled “little green turds,” they were, more often than not, still quite tasty—proving that even imperfect results can have their merits (with just a little bit of positive spin).
